# Supplementary material for: Two-Component GW Calculations: Cubic Scaling Implementation and Comparison of Vertex-Corrected and Partially Self-Consistent GW Variants
Source: J Chem Theory Comput. 2023 Aug 18;19(17):5958–76. doi: 10.1021/acs.jctc.3c00512 (PMC10501001; doi:10.1021/acs.jctc.3c00512)
Supplement: Supplementary file 2 — ct3c00512_si_002.pdf [file ct3c00512_si_002.pdf]

Supporting information

Arno Förster,*,† Erik van Lenthe,*,‡ Edoardo Spadetto,‡ and Lucas Visscher†

†*Theoretical Chemistry, Vrije Universiteit, De Boelelaan 1083, NL, 1081HV, Amsterdam, The Netherlands*

‡*Software for Chemistry and Materials NV, NL, 1081HV, Amsterdam, The Netherlands*

E-mail: a.t.l.foerster@vu.nl; vanlenthe@scm.com

A Accuracy of analytical continuation

In this section we compare our scalar relativistic G_0W_0@PBE/TZ3P results with AC of the self-energy to full-frequency calculations for the 67 systems in SOC81*. The individual results are shown in table 1

Table 1: G_0W_0@PBE/TZ3P scalar relativistic first IPs in the SOC81* set with full-frequency and AC implementation of the self-energy. All values are in eV.

Name	full-frequency	analytical continuation	Δ
Al_2Br_6	9.948	9.942	−0.006
$AlBr_3$	10.090	10.095	0.005
AlI_3	9.046	9.057	0.011
$AsBr_3$	9.383	9.393	0.010
$AsCl_3$	10.043	10.058	0.015
AsF_3	12.081	12.079	−0.002
AsF_5	14.269	14.275	0.006
AsH_3	9.955	9.978	0.023
AsI_3	8.623	8.637	0.014
Br_2	9.921	9.923	0.002
BrCl	10.303	10.304	0.001
$C_{10}H_{10}Ru$	6.472	6.475	0.003
C_2H_2Se	8.116	8.116	0.000
C_2H_6Cd	8.510	8.496	−0.015
C_2H_6Hg	8.686	8.682	−0.005
C_2H_6Se	7.767	7.772	0.005
C_2H_6Zn	9.078	9.070	−0.008

Continued on next page

Name	full-frequency	analytical continuation	Δ
C_2HBrO	8.677	8.677	0.000
C_4H_4Se	8.365	8.367	0.002
CF_3I	9.373	9.383	0.010
CH_3HgBr	8.736	8.747	0.011
CH_3HgCl	9.786	9.796	0.010
CH_3HgI	10.407	10.420	0.013
CH_3I	9.043	9.054	0.011
CI_4	10.133	10.140	0.007
$CaBr_2$	9.308	9.319	0.011
CaI_2	9.795	9.816	0.021
$CdBr_2$	8.721	8.730	0.009
$CdCl_2$	9.199	9.206	0.007
CdI_2	8.551	8.541	−0.010
CsF	7.897	7.853	−0.044
$HgCl_2$	10.401	10.420	0.019
I_2	9.007	9.005	−0.002
IBr	9.393	9.395	0.002
ICl	9.677	9.678	0.001
IF	10.180	10.183	0.003
Kr_2	12.859	12.859	0.000
KrF_2	12.295	12.311	0.016
$LaBr_3$	9.426	9.439	0.013
$LaCl_3$	10.159	10.151	−0.008
LiBr	8.501	8.510	0.009
LiI	7.961	7.984	0.023
$MgBr_2$	10.051	10.062	0.011
MgI_2	9.227	9.244	0.017
MoC_6O_6	8.022	8.019	−0.003
OsO_4	11.569	11.579	0.010
PBr_3	9.223	9.235	0.012
$POBr_3$	10.212	10.218	0.006
RuO_4	11.288	11.296	0.008
$SOBr_2$	8.681	8.684	0.003
$SPBr_3$	10.840	10.843	0.003
$SeCl_2$	11.321	11.329	0.008
SeO_2	9.554	9.560	0.006
$SiBrF_3$	9.802	9.829	0.027
SiH_3I	9.000	9.008	0.008
$SrBr_2$	9.079	9.082	0.003
$SrCl_2$	9.507	9.513	0.006
SrI_2	8.496	8.494	−0.002
$TiBr_4$	9.619	9.598	−0.021
TiI_4	8.585	8.589	0.004
$ZnBr_2$	10.101	10.109	0.008
$ZnCl_2$	10.820	10.848	0.027
ZnF_2	12.546	12.553	0.007
ZnI_2	9.254	9.264	0.010
$ZrBr_4$	9.830	9.859	0.029
$ZrCl_4$	10.800	10.817	0.017
ZrI_4	8.810	8.790	−0.020

B QP energies included in the benchmark

All QP energies calculated in this work are tabulated here. All values are in eV. The basis set limit extrapolation has been performed as stated in the main text. The 2C QP energies and the $G3W2$ corrected QP energies can be calculated form the values in the table by adding the terms Δ_{SO} and Δ_{G3W2}, respectively.

Table 2: All G_0W_0@PBE QP energies calculated in this work for the systems included in the benchmark: ADF results (TZ3P, QZ6P, extrapolated), BAND results (TZ3P+, QZ6P+, extrapolated), spin-orbit correction, and $G3W2$ correction, both calculated using ADF with the QZ6P basis set. All values are in eV.

	ADF			BAND				
Name	TZ3P	QZ6P	extra	TZ3P+	QZ6P+	extra	Δ_{SO}	Δ_{G3W2}
Al_2Br_6	9.94	10.09	10.26	10.17	10.24	10.32	−0.02	0.08
$AlBr_3$	10.09	10.25	10.43	10.32	10.38	10.47	−0.03	0.08
AlI_3	9.05	9.09	9.16	9.17	9.23	9.32	−0.13	0.06
$AsBr_3$	9.38	9.56	9.77	9.62	9.70	9.79	−0.03	0.04
$AsCl_3$	10.04	10.27	10.52	10.13	10.31	10.53	0.00	0.01
AsF_3	12.08	12.28	12.49	12.22	12.30	12.38	0.00	0.04
AsF_5	14.27	14.36	14.45	14.27	14.37	14.48	−0.01	0.22
AsH_3	9.96	10.20	10.47	10.11	10.27	10.42	0.00	−0.01
AsI_3	8.62	8.65	8.71	8.76	8.80	8.86	−0.16	0.10
Br_2	9.92	10.08	10.26	10.18	10.23	10.29	−0.13	0.07
BrCl	10.30	10.47	10.66	10.45	10.56	10.69	−0.10	0.06
$C_{10}H_{10}Ru$	6.47	6.53	6.60	6.62	6.74	6.89	−0.06	0.01
C_2H_2Se	8.12	8.26	8.43	8.23	8.34	8.48	−0.01	−0.04
C_2H_6Cd	8.51	8.60	8.69	8.51	8.67	8.86	0.00	−0.04
C_2H_6Hg	8.69	8.78	8.89	8.68	8.88	9.10	0.02	−0.06
C_2H_6Se	7.77	7.91	8.08	7.94	8.03	8.14	0.00	−0.03
C_2H_6Zn	9.08	9.19	9.32	9.12	9.26	9.42	0.00	−0.05
C_2HBrO	8.68	8.81	8.96	8.76	8.89	9.04	−0.01	−0.05
C_4H_4Se	8.37	8.48	8.61	8.37	8.53	8.72	0.00	−0.08
CF_3I	10.13	10.17	10.22	10.25	10.30	10.37	−0.24	0.05
CH_3HgBr	9.31	9.39	9.49	9.51	9.54	9.56	−0.09	0.09
CH_3HgCl	9.79	9.90	10.01	9.80	9.93	10.09	−0.02	0.08
CH_3HgI	8.72	8.73	8.74	8.81	8.83	8.85	−0.19	0.06
CH_3I	9.20	9.23	9.27	9.31	9.36	9.42	−0.23	0.02
CI_4	8.55	8.60	8.68	8.68	8.75	8.86	−0.23	0.07
$CaBr_2$	9.37	9.49	9.64	9.57	9.61	9.66	−0.09	0.10
CaI_2	8.74	8.77	8.82	8.84	8.89	8.99	−0.19	0.08
$CdBr_2$	9.79	9.89	10.03	10.00	10.02	10.05	−0.10	0.11
$CdCl_2$	10.41	10.53	10.68	10.41	10.53	10.72	−0.03	0.11
CdI_2	9.04	9.07	9.10	9.15	9.20	9.29	−0.23	0.09
CsF	7.90	8.08	8.32	7.97	8.15	8.49	0.00	0.19
$HgCl_2$	10.40	10.54	10.70	10.49	10.56	10.66	−0.05	0.10
I_2	9.01	9.03	9.07	9.13	9.21	9.34	−0.29	0.05
IBr	9.39	9.47	9.58	9.57	9.62	9.70	−0.25	0.06
ICl	9.68	9.74	9.83	9.76	9.85	9.99	−0.25	0.05

Continued on next page

Name	ADF TZ3P	ADF QZ6P	ADF extra	BAND TZ3P+	BAND QZ6P+	BAND extra	Δ_{SO}	Δ_{G3W2}
IF	10.18	10.22	10.27	10.29	10.35	10.44	−0.30	0.07
Kr_2	12.86	13.05	13.28	13.17	13.22	13.28	−0.09	0.11
KrF_2	12.29	12.41	12.54	12.37	12.45	12.56	−0.05	0.19
$LaBr_3$	9.43	9.52	9.65	9.65	9.71	9.80	−0.03	0.12
$LaCl_3$	10.16	10.29	10.45	10.18	10.34	10.58	−0.01	0.09
LiBr	8.50	8.62	8.74	8.68	8.73	8.79	−0.09	0.10
LiI	7.96	7.99	8.04	8.07	8.08	8.10	−0.20	0.08
$MgBr_2$	10.05	10.18	10.33	10.26	10.31	10.37	−0.10	0.11
MgI_2	9.23	9.28	9.38	9.33	9.41	9.52	−0.22	0.08
MoC_6O_6	8.02	8.16	8.35	8.13	8.31	8.55	−0.03	−0.03
OsO_4	11.57	11.57	11.58	11.47	11.63	11.82	0.00	0.15
PBr_3	9.22	9.36	9.51	9.39	9.47	9.56	−0.02	0.01
$POBr_3$	10.21	10.35	10.51	10.38	10.47	10.57	−0.06	0.10
RuO_4	11.29	11.32	11.36	11.29	11.37	11.48	0.00	0.19
$SOBr_2$	9.80	9.94	10.09	9.95	10.03	10.12	−0.05	0.04
$SPBr_3$	9.00	9.14	9.30	9.06	9.25	9.47	−0.02	0.05
$SeCl_2$	8.68	8.87	9.08	8.82	8.96	9.13	−0.03	0.00
SeO_2	10.84	10.89	10.95	10.89	10.96	11.05	0.00	0.11
$SiBrF_3$	11.32	11.47	11.64	11.54	11.60	11.68	−0.11	0.07
SiH_3I	9.55	9.60	9.65	9.66	9.72	9.80	−0.22	0.03
$SrBr_2$	9.08	9.20	9.35	9.27	9.32	9.39	−0.09	0.11
$SrCl_2$	9.51	9.66	9.86	9.52	9.68	9.90	−0.01	0.08
SrI_2	8.50	8.52	8.57	8.59	8.67	8.79	−0.19	0.09
$TiBr_4$	9.62	9.75	9.90	9.80	9.86	9.93	−0.07	0.20
TiI_4	8.52	8.59	8.71	8.69	8.72	8.77	−0.15	0.19
$ZnBr_2$	10.10	10.23	10.39	10.32	10.35	10.39	−0.11	0.10
$ZnCl_2$	10.82	10.99	11.17	10.84	10.98	11.19	−0.03	0.10
ZnF_2	12.55	12.60	12.66	12.57	12.57	12.57	−0.01	0.24
ZnI_2	9.25	9.30	9.37	9.37	9.44	9.55	−0.23	0.08
$ZrBr_4$	9.83	9.97	10.14	10.04	10.11	10.20	−0.05	0.13
$ZrCl_4$	10.80	10.95	11.13	10.78	10.98	11.26	−0.01	0.12
ZrI_4	8.79	8.85	8.96	8.89	9.01	9.20	−0.16	0.10

Table 3: All G_0W_0@PBE0 QP energies calculated in this work for the systems included in the benchmark: ADF results (TZ3P, QZ6P, extrapolated), BAND results (TZ3P+, QZ6P+, extrapolated), spin-orbit correction, and $G3W2$ correction, both calculated using ADF with the QZ6P basis set. All values are in eV.

Name	ADF TZ3P	ADF QZ6P	ADF extra	BAND TZ3P+	BAND QZ6P+	BAND extra	Δ_{SO}	Δ_{G3W2}
Al_2Br_6	10.33	10.50	10.69	10.57	10.64	10.73	−0.03	0.09
$AlBr_3$	10.45	10.62	10.81	10.68	10.76	10.85	−0.04	0.09
AlI_3	9.39	9.43	9.49	9.51	9.57	9.67	−0.15	0.07
$AsBr_3$	9.68	9.90	10.14	9.94	10.03	10.14	−0.05	0.03
$AsCl_3$	10.37	10.62	10.89	10.47	10.66	10.89	0.00	0.00
AsF_3	12.43	12.63	12.85	12.59	12.69	12.80	0.00	0.03
AsF_5	15.02	15.13	15.26	15.02	15.15	15.31	−0.01	0.26
AsH_3	10.15	10.33	10.53	10.32	10.43	10.54	0.00	−0.02
AsI_3	8.95	9.05	9.22	9.11	9.20	9.33	−0.22	0.05
Br_2	10.16	10.33	10.53	10.42	10.48	10.55	−0.15	0.07

Continued on next page

Name	ADF TZ3P	ADF QZ6P	ADF extra	BAND TZ3P+	BAND QZ6P+	BAND extra	Δ_{SO}	Δ_{G3W2}
BrCl	10.55	10.74	10.95	10.71	10.83	10.98	−0.11	0.06
$C_{10}H_{10}Ru$	6.70	6.79	6.91	6.90	7.04	7.21	−0.09	0.05
C_2H_2Se	8.32	8.48	8.66	8.44	8.57	8.72	−0.01	−0.04
C_2H_6Cd	8.74	8.84	8.95	8.74	8.92	9.16	0.00	−0.03
C_2H_6Hg	8.87	8.97	9.09	8.88	9.08	9.30	0.03	−0.05
C_2H_6Se	7.97	8.14	8.33	8.14	8.25	8.38	−0.01	−0.03
C_2H_6Zn	9.31	9.44	9.57	9.35	9.51	9.70	0.00	−0.04
C_2HBrO	8.96	9.10	9.27	9.04	9.19	9.36	−0.01	−0.06
C_4H_4Se	8.62	8.73	8.88	8.62	8.78	8.98	0.00	−0.08
CF_3I	10.43	10.46	10.50	10.55	10.60	10.66	−0.27	0.06
CH_3HgBr	9.63	9.77	9.92	9.83	9.90	9.98	−0.11	0.11
CH_3HgCl	10.20	10.36	10.53	10.19	10.39	10.64	−0.03	0.11
CH_3HgI	8.99	9.03	9.06	9.08	9.15	9.23	−0.23	0.08
CH_3I	9.39	9.43	9.48	9.51	9.56	9.63	−0.26	0.03
CI_4	8.88	8.93	9.02	9.02	9.11	9.26	−0.22	0.07
$CaBr_2$	9.76	9.90	10.07	9.97	10.03	10.10	−0.12	0.12
CaI_2	9.10	9.14	9.22	9.21	9.24	9.29	−0.24	0.10
$CdBr_2$	10.15	10.29	10.46	10.37	10.42	10.49	−0.13	0.13
$CdCl_2$	10.83	10.99	11.18	10.85	11.00	11.23	−0.03	0.14
CdI_2	9.37	9.40	9.46	9.49	9.54	9.62	−0.26	0.10
CsF	8.64	8.87	9.18	8.64	8.93	9.51	0.00	0.25
$HgCl_2$	10.79	10.96	11.14	10.89	10.98	11.08	−0.06	0.12
I_2	9.22	9.26	9.33	9.35	9.47	9.64	−0.31	0.05
IBr	9.62	9.70	9.83	9.79	9.86	9.95	−0.27	0.06
ICl	9.90	9.98	10.09	9.99	10.09	10.24	−0.27	0.05
IF	10.41	10.46	10.54	10.53	10.59	10.66	−0.32	0.07
Kr_2	13.09	13.32	13.59	13.40	13.48	13.57	−0.11	0.11
KrF_2	12.97	13.12	13.28	13.07	13.16	13.28	−0.07	0.22
$LaBr_3$	9.91	10.06	10.26	10.15	10.22	10.32	−0.08	0.15
$LaCl_3$	10.65	10.83	11.04	10.68	10.87	11.17	−0.01	0.12
LiBr	8.83	8.97	9.12	9.03	9.09	9.16	−0.11	0.12
LiI	8.24	8.29	8.36	8.35	8.41	8.48	−0.23	0.09
$MgBr_2$	10.42	10.57	10.75	10.64	10.70	10.79	−0.12	0.12
MgI_2	9.57	9.62	9.72	9.68	9.76	9.87	−0.25	0.09
MoC_6O_6	8.23	8.38	8.58	8.34	8.54	8.78	−0.04	−0.03
OsO_4	12.20	12.22	12.25	12.17	12.29	12.44	−0.02	0.23
PBr_3	9.53	9.68	9.84	9.70	9.79	9.89	−0.03	0.00
$POBr_3$	10.66	10.80	10.96	10.90	10.97	11.05	−0.10	0.11
RuO_4	11.99	12.03	12.08	11.99	12.10	12.24	0.00	0.32
$SOBr_2$	10.20	10.35	10.52	10.34	10.45	10.58	−0.06	0.05
$SPBr_3$	9.34	9.50	9.68	9.40	9.57	9.77	−0.02	0.07
$SeCl_2$	8.96	9.16	9.39	9.11	9.26	9.45	−0.02	0.00
SeO_2	11.35	11.45	11.57	11.42	11.52	11.65	0.00	0.14
$SiBrF_3$	11.63	11.78	11.96	11.85	11.92	12.00	−0.13	0.08
SiH_3I	9.79	9.83	9.90	9.89	9.97	10.06	−0.25	0.04
$SrBr_2$	9.44	9.58	9.76	9.65	9.71	9.79	−0.12	0.13
$SrCl_2$	9.91	10.09	10.31	9.91	10.11	10.39	−0.01	0.10
SrI_2	8.82	8.87	8.96	8.93	8.99	9.07	−0.23	0.11
$TiBr_4$	10.13	10.30	10.49	10.36	10.44	10.54	−0.08	0.23
TiI_4	9.03	9.08	9.18	9.16	9.24	9.35	−0.19	0.21
$ZnBr_2$	10.44	10.60	10.78	10.66	10.73	10.81	−0.13	0.11
$ZnCl_2$	11.22	11.40	11.61	11.23	11.40	11.66	−0.03	0.12
ZnF_2	13.20	13.27	13.35	13.22	13.25	13.30	−0.02	0.30

Continued on next page

Name	ADF TZ3P	ADF QZ6P	ADF extra	BAND TZ3P+	BAND QZ6P+	BAND extra	Δ_{SO}	Δ_{G3W2}
ZnI_2	9.56	9.61	9.69	9.69	9.76	9.89	−0.27	0.09
$ZrBr_4$	10.33	10.49	10.70	10.55	10.64	10.75	−0.08	0.15
$ZrCl_4$	11.37	11.54	11.74	11.37	11.56	11.82	−0.02	0.16
ZrI_4	9.22	9.28	9.37	9.35	9.44	9.57	−0.19	0.12

Table 4: All G_0W_0@BHLYP QP energies calculated in this work for the systems included in the benchmark: ADF results (TZ3P, QZ6P, extrapolated), BAND results (TZ3P+, QZ6P+, extrapolated), spin-orbit correction, and $G3W2$ correction, both calculated using ADF with the QZ6P basis set. All values are in eV.

Name	ADF TZ3P	ADF QZ6P	ADF extra	BAND TZ3P+	BAND QZ6P+	BAND extra	Δ_{SO}	Δ_{G3W2}
Al_2Br_6	10.61	10.79	10.98	10.85	10.93	11.02	−0.04	0.10
$AlBr_3$	10.69	10.87	11.07	10.93	11.01	11.10	−0.05	0.10
AlI_3	9.63	9.67	9.75	9.75	9.82	9.92	−0.17	0.07
$AsBr_3$	9.93	10.14	10.39	10.19	10.28	10.39	−0.06	0.03
$AsCl_3$	10.62	10.88	11.15	10.74	10.93	11.15	0.00	0.00
AsF_3	12.72	12.96	13.21	12.90	13.01	13.14	0.00	0.03
AsF_5	15.48	15.62	15.77	15.49	15.64	15.83	−0.01	0.30
AsH_3	10.31	10.52	10.74	10.47	10.59	10.70	0.00	−0.01
AsI_3	9.19	9.30	9.47	9.35	9.44	9.58	−0.24	0.04
Br_2	10.34	10.52	10.72	10.60	10.67	10.75	−0.17	0.08
BrCl	10.74	10.94	11.16	10.91	11.03	11.18	−0.12	0.07
$C_{10}H_{10}Ru$	7.02	7.12	7.25	7.23	7.37	7.55	−0.11	0.09
C_2H_2Se	8.48	8.64	8.83	8.60	8.73	8.89	−0.01	−0.04
C_2H_6Cd	8.91	9.03	9.15	8.93	9.11	9.33	0.00	−0.02
C_2H_6Hg	9.03	9.15	9.28	8.99	9.24	9.53	0.05	−0.05
C_2H_6Se	8.14	8.33	8.53	8.32	8.44	8.57	0.00	−0.02
C_2H_6Zn	9.49	9.62	9.77	9.54	9.70	9.89	0.00	−0.02
C_2HBrO	9.19	9.34	9.51	9.28	9.43	9.60	−0.01	−0.06
C_4H_4Se	8.80	8.92	9.07	8.81	8.97	9.16	0.00	−0.08
CF_3I	10.71	10.74	10.79	10.83	10.89	10.97	−0.30	0.07
CH_3HgBr	9.85	9.99	10.16	10.06	10.13	10.21	−0.13	0.13
CH_3HgCl	10.45	10.63	10.82	10.43	10.65	10.92	−0.03	0.13
CH_3HgI	9.19	9.23	9.28	9.29	9.36	9.45	−0.26	0.09
CH_3I	9.55	9.60	9.67	9.67	9.73	9.82	−0.29	0.04
CI_4	9.14	9.19	9.28	9.28	9.38	9.53	−0.22	0.08
$CaBr_2$	9.98	10.13	10.30	10.20	10.26	10.34	−0.14	0.15
CaI_2	9.31	9.36	9.44	9.42	9.47	9.54	−0.27	0.12
$CdBr_2$	10.38	10.53	10.71	10.60	10.66	10.74	−0.14	0.15
$CdCl_2$	11.09	11.27	11.48	11.11	11.28	11.54	−0.04	0.16
CdI_2	9.59	9.63	9.70	9.71	9.77	9.86	−0.29	0.11
CsF	9.00	9.22	9.51	8.99	9.26	9.79	0.00	0.28
$HgCl_2$	11.05	11.22	11.42	11.16	11.24	11.36	−0.06	0.14
I_2	9.40	9.45	9.53	9.54	9.63	9.78	−0.33	0.06
IBr	9.80	9.89	10.02	9.98	10.04	10.14	−0.28	0.06
ICl	10.10	10.18	10.29	10.19	10.30	10.47	−0.28	0.06
IF	10.63	10.68	10.75	10.75	10.81	10.89	−0.33	0.07
Kr_2	13.27	13.50	13.79	13.55	13.67	13.82	−0.13	0.12
KrF_2	13.56	13.59	13.61	13.60	13.76	13.96	−0.07	0.24

Continued on next page

Name	ADF TZ3P	ADF QZ6P	ADF extra	BAND TZ3P+	BAND QZ6P+	BAND extra	Δ_{SO}	Δ_{G3W2}
$LaBr_3$	10.20	10.36	10.55	10.43	10.51	10.61	−0.09	0.18
$LaCl_3$	10.98	11.16	11.38	11.00	11.20	11.51	−0.01	0.15
LiBr	9.01	9.15	9.31	9.22	9.29	9.36	−0.13	0.13
LiI	8.41	8.46	8.53	8.52	8.58	8.66	−0.26	0.10
$MgBr_2$	10.64	10.79	10.98	10.85	10.93	11.02	−0.14	0.14
MgI_2	9.78	9.83	9.93	9.89	9.97	10.08	−0.28	0.10
MoC_6O_6	8.49	8.65	8.86	8.62	8.81	9.06	−0.05	−0.01
OsO_4	12.63	12.66	12.69	12.59	12.73	12.89	−0.06	0.33
PBr_3	9.79	9.94	10.10	9.95	10.04	10.15	−0.03	0.00
$POBr_3$	10.97	11.15	11.34	11.22	11.29	11.38	−0.13	0.11
RuO_4	12.45	12.50	12.57	12.45	12.57	12.73	−0.01	0.48
$SOBr_2$	10.47	10.63	10.80	10.62	10.73	10.86	−0.06	0.05
$SPBr_3$	9.57	9.74	9.92	9.63	9.81	10.03	−0.03	0.08
$SeCl_2$	9.20	9.41	9.64	9.35	9.50	9.69	−0.01	0.01
SeO_2	11.67	11.78	11.91	11.74	11.86	12.01	0.00	0.16
$SiBrF_3$	11.86	12.02	12.20	12.09	12.15	12.23	−0.14	0.09
SiH_3I	9.97	10.02	10.09	10.08	10.16	10.26	−0.28	0.05
$SrBr_2$	9.65	9.80	9.99	9.87	9.93	10.02	−0.14	0.15
$SrCl_2$	10.16	10.35	10.59	10.16	10.37	10.66	−0.01	0.12
SrI_2	9.03	9.08	9.17	9.14	9.20	9.29	−0.27	0.12
$TiBr_4$	10.49	10.66	10.86	10.72	10.80	10.90	−0.10	0.28
TiI_4	9.35	9.41	9.51	9.48	9.56	9.68	−0.22	0.25
$ZnBr_2$	10.66	10.82	11.01	10.89	10.96	11.04	−0.15	0.13
$ZnCl_2$	11.47	11.66	11.87	11.48	11.67	11.95	−0.04	0.14
ZnF_2	13.51	13.62	13.75	13.53	13.62	13.74	−0.02	0.35
ZnI_2	9.78	9.83	9.92	9.90	9.99	10.12	−0.30	0.10
$ZrBr_4$	10.66	10.83	11.04	10.90	10.98	11.09	−0.10	0.18
$ZrCl_4$	11.74	11.92	12.13	11.75	11.94	12.22	−0.02	0.18
ZrI_4	9.54	9.59	9.68	9.66	9.75	9.90	−0.22	0.14

Table 5: All ev$GW0$@PBE0 QP energies calculated in this work for the systems included in the benchmark: ADF results (TZ3P, QZ6P, extrapolated), BAND results (TZ3P+, QZ6P+, extrapolated), spin-orbit correction, and $G3W2$ correction, both calculated using ADF with the QZ6P basis set. All values are in eV.

Name	ADF TZ3P	ADF QZ6P	ADF extra	BAND TZ3P+	BAND QZ6P+	BAND extra	Δ_{SO}	Δ_{G3W2}
Al_2Br_6	10.46	10.67	10.90	10.73	10.83	10.96	−0.04	0.09
$AlBr_3$	10.57	10.78	11.01	10.84	10.95	11.07	−0.04	0.08
AlI_3	9.49	9.55	9.64	9.63	9.72	9.84	−0.15	0.06
$AsBr_3$	9.75	10.00	10.29	10.04	10.16	10.30	−0.04	0.02
$AsCl_3$	10.44	10.73	11.05	10.55	10.78	11.05	0.00	0.00
AsF_3	12.56	12.82	13.11	12.73	12.87	13.03	0.00	0.03
AsF_5	15.34	15.50	15.68	15.34	15.53	15.75	−0.01	0.26
AsH_3	10.25	10.46	10.70	10.42	10.56	10.70	0.00	−0.02
AsI_3	9.02	9.21	9.52	9.20	9.31	9.47	−0.27	0.10
Br_2	10.25	10.46	10.70	10.54	10.63	10.73	−0.15	0.06
BrCl	10.64	10.88	11.13	10.82	10.97	11.16	−0.12	0.06
$C_{10}H_{10}Ru$	6.78	6.93	7.13	7.01	7.15	7.32	−0.09	0.04
C_2H_2Se	8.38	8.57	8.80	8.51	8.67	8.87	−0.01	−0.04

Continued on next page

Name	ADF TZ3P	ADF QZ6P	ADF extra	BAND TZ3P+	BAND QZ6P+	BAND extra	Δ_{SO}	Δ_{G3W2}
C_2H_6Cd	8.86	9.01	9.17	8.89	9.08	9.31	0.00	−0.04
C_2H_6Hg	8.96	9.11	9.26	8.93	9.20	9.51	0.03	−0.06
C_2H_6Se	8.07	8.28	8.52	8.26	8.40	8.56	0.00	−0.03
C_2H_6Zn	9.46	9.62	9.79	9.50	9.68	9.89	0.00	−0.04
C_2HBrO	9.03	9.21	9.41	9.13	9.30	9.51	−0.01	−0.06
C_4H_4Se	8.68	8.84	9.03	8.69	8.89	9.13	0.00	−0.08
CF_3I	10.52	10.57	10.64	10.65	10.72	10.82	−0.28	0.06
CH_3HgBr	9.77	9.95	10.16	10.00	10.10	10.22	−0.12	0.11
CH_3HgCl	10.37	10.58	10.82	10.32	10.61	10.96	−0.03	0.11
CH_3HgI	9.10	9.16	9.24	9.20	9.30	9.44	−0.24	0.08
CH_3I	9.48	9.54	9.63	9.61	9.68	9.79	−0.27	0.03
CI_4	8.97	9.04	9.16	9.13	9.24	9.41	−0.22	0.07
$CaBr_2$	9.89	10.08	10.29	10.13	10.22	10.33	−0.12	0.12
CaI_2	9.20	9.27	9.38	9.33	9.39	9.48	−0.24	0.10
$CdBr_2$	10.28	10.47	10.70	10.54	10.63	10.74	−0.13	0.12
$CdCl_2$	11.00	11.21	11.46	11.02	11.23	11.53	−0.03	0.13
CdI_2	9.49	9.54	9.63	9.62	9.70	9.82	−0.27	0.10
CsF	8.98	9.24	9.58	8.98	9.29	9.91	0.01	0.24
$HgCl_2$	10.94	11.16	11.40	11.04	11.18	11.35	−0.06	0.12
I_2	9.29	9.35	9.46	9.44	9.57	9.77	−0.32	0.05
IBr	9.70	9.80	9.96	9.89	9.98	10.10	−0.27	0.05
ICl	9.97	10.08	10.24	10.08	10.21	10.39	−0.28	0.05
IF	10.49	10.56	10.66	10.62	10.70	10.81	−0.33	0.06
Kr_2	13.21	13.45	13.75	13.55	13.65	13.77	−0.12	0.12
KrF_2	13.26	13.46	13.68	13.36	13.52	13.70	−0.08	0.21
$LaBr_3$	10.04	10.24	10.49	10.32	10.42	10.55	−0.08	0.14
$LaCl_3$	10.80	11.02	11.28	10.83	11.07	11.43	−0.01	0.11
LiBr	8.97	9.15	9.35	9.21	9.30	9.40	−0.12	0.12
LiI	8.36	8.43	8.53	8.49	8.57	8.68	−0.24	0.09
$MgBr_2$	10.56	10.75	10.98	10.81	10.91	11.03	−0.13	0.12
MgI_2	9.69	9.75	9.87	9.82	9.91	10.05	−0.26	0.09
MoC_6O_6	8.21	8.40	8.65	8.34	8.57	8.87	−0.04	−0.04
OsO_4	12.33	12.40	12.49	12.29	12.49	12.72	−0.02	0.21
PBr_3	9.60	9.78	9.99	9.79	9.90	10.04	−0.03	0.00
$POBr_3$	10.77	10.97	11.20	11.04	11.14	11.25	−0.12	0.09
RuO_4	12.12	12.20	12.32	12.13	12.30	12.51	0.00	0.28
$SOBr_2$	10.28	10.47	10.68	10.44	10.58	10.75	−0.05	0.04
$SPBr_3$	9.44	9.63	9.85	9.52	9.72	9.96	−0.02	0.06
$SeCl_2$	9.04	9.29	9.56	9.20	9.38	9.62	−0.02	0.00
SeO_2	11.54	11.67	11.84	11.61	11.76	11.94	−0.01	0.13
$SiBrF_3$	11.74	11.92	12.13	11.98	12.07	12.17	−0.13	0.08
SiH_3I	9.88	9.95	10.04	10.00	10.10	10.23	−0.25	0.04
$SrBr_2$	9.56	9.75	9.99	9.81	9.90	10.02	−0.12	0.12
$SrCl_2$	10.06	10.28	10.56	10.05	10.30	10.65	−0.01	0.10
SrI_2	8.93	9.00	9.12	9.06	9.14	9.26	−0.24	0.10
$TiBr_4$	10.26	10.48	10.74	10.53	10.65	10.79	−0.09	0.19
TiI_4	9.13	9.21	9.35	9.29	9.39	9.55	−0.20	0.16
$ZnBr_2$	10.57	10.78	11.02	10.83	10.93	11.05	−0.13	0.11
$ZnCl_2$	11.37	11.62	11.88	11.39	11.62	11.95	−0.04	0.11
ZnF_2	13.56	13.72	13.91	13.59	13.71	13.87	−0.02	0.29
ZnI_2	9.67	9.74	9.87	9.81	9.92	10.08	−0.28	0.08
$ZrBr_4$	10.46	10.67	10.92	10.73	10.84	10.98	−0.08	0.14
$ZrCl_4$	11.53	11.74	11.99	11.53	11.76	12.10	−0.02	0.14

Continued on next page

Name	ADF TZ3P	ADF QZ6P	ADF extra	BAND TZ3P+	BAND QZ6P+	BAND extra	Δ_{SO}	Δ_{G3W2}
ZrI_4	9.33	9.40	9.53	9.47	9.59	9.77	−0.20	0.11

Table 6: All evGW@PBE0 QP energies calculated in this work for the systems included in the benchmark: ADF results (TZ3P, QZ6P, extrapolated), BAND results (TZ3P+, QZ6P+, extrapolated), spin-orbit correction, and $G3W2$ correction, both calculated using ADF with the QZ6P basis set. All values are in eV.

Name	ADF TZ3P	ADF QZ6P	ADF extra	BAND TZ3P+	BAND QZ6P+	BAND extra	Δ_{SO}	Δ_{G3W2}
Al_2Br_6	10.67	10.86	11.07	10.94	11.02	11.13	−0.04	0.11
$AlBr_3$	10.78	10.97	11.19	11.04	11.13	11.23	−0.04	0.10
AlI_3	9.66	9.70	9.78	9.80	9.87	9.99	−0.16	0.08
$AsBr_3$	9.90	10.14	10.42	10.19	10.30	10.42	−0.04	0.03
$AsCl_3$	10.59	10.87	11.17	10.70	10.91	11.17	0.00	0.00
AsF_3	12.76	12.99	13.25	12.92	13.05	13.21	0.00	0.04
AsF_5	15.76	15.91	16.08	15.76	15.93	16.14	−0.01	0.34
AsH_3	10.35	10.57	10.81	10.53	10.65	10.78	0.00	−0.02
AsI_3	9.16	9.37	9.74	9.33	9.44	9.59	−0.31	0.12
Br_2	10.41	10.61	10.82	10.69	10.77	10.86	−0.16	0.08
BrCl	10.81	11.03	11.27	10.98	11.12	11.29	−0.12	0.07
$C_{10}H_{10}Ru$	6.93	7.11	7.32	7.17	7.33	7.53	−0.10	0.09
C_2H_2Se	8.49	8.67	8.89	8.62	8.78	8.96	−0.01	−0.04
C_2H_6Cd	9.01	9.13	9.27	9.03	9.21	9.44	0.01	−0.01
C_2H_6Hg	9.09	9.23	9.38	9.06	9.31	9.61	0.03	−0.05
C_2H_6Se	8.21	8.41	8.63	8.39	8.52	8.67	−0.01	−0.02
C_2H_6Zn	9.62	9.77	9.93	9.67	9.84	10.04	0.00	−0.02
C_2HBrO	9.17	9.34	9.53	9.26	9.43	9.63	−0.01	−0.06
C_4H_4Se	8.81	8.96	9.14	8.82	9.01	9.24	0.00	−0.07
CF_3I	10.65	10.69	10.75	10.78	10.84	10.92	−0.29	0.07
CH_3HgBr	9.98	10.15	10.33	10.21	10.30	10.41	−0.12	0.14
CH_3HgCl	10.61	10.81	11.04	10.59	10.84	11.13	−0.04	0.15
CH_3HgI	9.27	9.31	9.37	9.37	9.46	9.58	−0.25	0.10
CH_3I	9.61	9.66	9.73	9.74	9.81	9.90	−0.28	0.04
CI_4	9.13	9.18	9.28	9.28	9.39	9.55	−0.23	0.08
$CaBr_2$	10.11	10.28	10.48	10.35	10.43	10.52	−0.13	0.16
CaI_2	9.38	9.43	9.52	9.50	9.56	9.64	−0.26	0.12
$CdBr_2$	10.51	10.68	10.89	10.76	10.83	10.93	−0.14	0.16
$CdCl_2$	11.27	11.46	11.68	11.27	11.46	11.74	−0.04	0.18
CdI_2	9.66	9.71	9.78	9.80	9.87	9.97	−0.28	0.12
CsF	9.44	9.68	10.00	9.43	9.73	10.33	−0.01	0.35
$HgCl_2$	11.19	11.39	11.61	11.29	11.41	11.55	−0.07	0.15
I_2	9.42	9.46	9.55	9.57	9.69	9.88	−0.32	0.06
IBr	9.83	9.93	10.07	10.02	10.10	10.21	−0.28	0.07
ICl	10.11	10.21	10.35	10.21	10.33	10.51	−0.29	0.06
IF	10.62	10.68	10.77	10.75	10.83	10.94	−0.34	0.07
Kr_2	13.39	13.62	13.90	13.73	13.81	13.90	−0.12	0.13
KrF_2	13.64	13.83	14.04	13.73	13.89	14.08	−0.09	0.30
$LaBr_3$	10.31	10.48	10.69	10.57	10.65	10.76	−0.09	0.18
$LaCl_3$	11.06	11.27	11.52	11.08	11.31	11.66	−0.02	0.16
LiBr	9.16	9.34	9.53	9.40	9.48	9.57	−0.13	0.15

Continued on next page

Name	ADF TZ3P	QZ6P	extra	BAND TZ3P+	QZ6P+	extra	Δ_{SO}	Δ_{G3W2}
LiI	8.51	8.56	8.64	8.64	8.71	8.81	−0.25	0.11
$MgBr_2$	10.77	10.95	11.16	11.02	11.10	11.21	−0.13	0.15
MgI_2	9.86	9.91	10.02	9.99	10.07	10.20	−0.27	0.11
MoC_6O_6	8.32	8.51	8.76	8.44	8.66	8.95	−0.04	−0.03
OsO_4	12.64	12.70	12.77	12.61	12.78	12.99	−0.02	0.36
PBr_3	9.74	9.91	10.10	9.92	10.03	10.15	−0.03	0.00
$POBr_3$	10.97	11.16	11.37	11.24	11.32	11.42	−0.11	0.12
RuO_4	12.44	12.51	12.61	12.45	12.61	12.81	0.01	0.49
$SOBr_2$	10.46	10.63	10.84	10.62	10.75	10.90	−0.04	0.06
$SPBr_3$	9.62	9.80	10.00	9.70	9.89	10.11	−0.02	0.09
$SeCl_2$	9.19	9.42	9.68	9.35	9.51	9.74	−0.02	0.00
SeO_2	11.81	11.93	12.09	11.87	12.01	12.19	0.00	0.20
$SiBrF_3$	11.91	12.08	12.27	12.15	12.22	12.32	−0.13	0.10
SiH_3I	10.03	10.08	10.16	10.15	10.23	10.35	−0.27	0.05
$SrBr_2$	9.79	9.96	10.18	10.03	10.11	10.21	−0.13	0.16
$SrCl_2$	10.28	10.52	10.80	10.29	10.53	10.87	−0.01	0.13
SrI_2	9.11	9.16	9.26	9.24	9.31	9.41	−0.26	0.13
$TiBr_4$	10.51	10.70	10.92	10.78	10.86	10.95	−0.08	0.23
TiI_4	9.32	9.38	9.47	9.48	9.57	9.71	−0.20	0.20
$ZnBr_2$	10.78	10.97	11.20	11.04	11.12	11.23	−0.14	0.14
$ZnCl_2$	11.62	11.85	12.10	11.64	11.85	12.15	−0.04	0.15
ZnF_2	14.03	14.17	14.33	14.05	14.16	14.32	−0.02	0.40
ZnI_2	9.85	9.91	10.01	9.99	10.08	10.23	−0.29	0.10
$ZrBr_4$	10.70	10.89	11.12	10.96	11.06	11.18	−0.09	0.18
$ZrCl_4$	11.79	12.00	12.23	11.79	12.02	12.33	−0.02	0.19
ZrI_4	9.52	9.57	9.66	9.66	9.76	9.91	−0.21	0.14

Table 7: All qsGW QP energies calculated in this work for the systems included in the benchmark: ADF results (TZ3P, QZ6P, extrapolated), BAND results (TZ3P+, QZ6P+, extrapolated), spin-orbit correction, and $G3W2$ correction, both calculated using ADF with the QZ6P basis set. All values are in eV.

Name	ADF TZ3P	QZ6P	extra	BAND TZ3P+	QZ6P+	extra	Δ_{SO}	Δ_{G3W2}
Al_2Br_6	11.03	11.07	11.11	11.12	11.18	11.25	−0.01	0.11
$AlBr_3$	11.12	11.16	11.20	11.20	11.26	11.33	−0.03	0.10
AlI_3	9.89	9.91	9.96	9.98	9.95	9.90	−0.18	0.08
$AsBr_3$	10.27	10.35	10.44	10.39	10.46	10.53	−0.03	0.02
$AsCl_3$	10.92	11.07	11.24	10.99	11.16	11.37	0.03	−0.01
AsF_3	13.21	13.30	13.40	13.33	13.38	13.43	0.03	0.02
AsF_5	16.36	16.34	16.32	16.35	16.42	16.51	0.11	0.32
AsH_3	10.58	10.67	10.77	10.65	10.71	10.78	0.01	−0.01
AsI_3	9.43	9.49	9.58	9.54	9.54	9.55	−0.14	0.04
Br_2	10.73	10.77	10.82	10.83	10.88	10.93	−0.11	0.08
BrCl	11.11	11.18	11.26	11.16	11.27	11.40	−0.07	0.08
$C_{10}H_{10}Ru$	7.47	7.57	7.70	7.54	7.68	7.87	0.00	0.11
C_2H_2Se	8.71	8.80	8.91	8.75	8.86	9.00	0.04	−0.04
C_2H_6Cd	9.15	9.24	9.34	9.16	9.33	9.53	0.05	0.00
C_2H_6Hg	9.26	9.34	9.43	9.19	9.40	9.65	0.12	−0.03
C_2H_6Se	8.46	8.54	8.62	8.52	8.61	8.70	0.02	−0.01

Continued on next page

Name	ADF TZ3P	ADF QZ6P	ADF extra	BAND TZ3P+	BAND QZ6P+	BAND extra	Δ_{SO}	Δ_{G3W2}
C_2H_6Zn	9.77	9.84	9.92	9.80	9.91	10.05	0.05	−0.01
C_2HBrO	9.40	9.48	9.58	9.44	9.55	9.70	0.04	−0.06
C_4H_4Se	8.95	9.03	9.13	8.96	9.06	9.19	0.05	−0.06
CF_3I	10.96	10.96	10.97	11.03	11.00	10.97	−0.33	0.08
CH_3HgBr	10.30	10.33	10.37	10.34	10.42	10.51	−0.12	0.14
CH_3HgCl	10.90	10.99	11.09	10.85	11.06	11.31	0.01	0.15
CH_3HgI	9.47	9.51	9.56	9.50	9.52	9.56	−0.27	0.11
CH_3I	9.80	9.83	9.87	9.86	9.85	9.82	−0.31	0.05
CI_4	9.40	9.41	9.44	9.49	9.48	9.45	−0.18	0.08
$CaBr_2$	10.43	10.45	10.48	10.49	10.54	10.61	−0.13	0.16
CaI_2	9.59	9.63	9.69	9.66	9.59	9.48	−0.29	0.13
$CdBr_2$	10.84	10.86	10.89	10.91	10.97	11.04	−0.12	0.16
$CdCl_2$	11.54	11.63	11.75	11.55	11.72	11.96	0.01	0.17
CdI_2	9.89	9.91	9.93	9.96	9.93	9.89	−0.29	0.12
CsF	9.95	10.05	10.18	9.94	10.13	10.49	0.11	0.30
$HgCl_2$	11.49	11.58	11.68	11.50	11.66	11.85	0.00	0.16
I_2	9.64	9.66	9.68	9.73	9.70	9.66	−0.26	0.07
IBr	10.09	10.11	10.15	10.18	10.19	10.19	−0.14	0.07
ICl	10.35	10.38	10.44	10.40	10.44	10.49	−0.27	0.06
IF	10.89	10.89	10.89	10.96	10.94	10.89	−0.32	0.07
Kr_2	13.67	13.76	13.88	13.76	13.88	14.03	−0.13	0.13
KrF_2	14.18	14.20	14.22	14.23	14.29	14.37	0.00	0.28
$LaBr_3$	10.67	10.70	10.73	10.75	10.80	10.88	−0.08	0.19
$LaCl_3$	11.42	11.51	11.63	11.42	11.62	11.91	0.06	0.17
LiBr	9.44	9.46	9.49	9.49	9.55	9.61	−0.13	0.15
LiI	8.67	8.71	8.77	8.73	8.71	8.68	−0.27	0.11
$MgBr_2$	11.08	11.10	11.13	11.14	11.19	11.26	−0.13	0.15
MgI_2	10.05	10.09	10.15	10.12	10.11	10.08	−0.31	0.11
MoC_6O_6	8.62	8.76	8.95	8.64	8.85	9.11	−0.04	0.00
OsO_4	13.15	13.11	13.06	13.11	13.14	13.17	−0.20	0.40
PBr_3	10.08	10.11	10.14	10.15	10.21	10.27	0.00	0.00
$POBr_3$	11.38	11.41	11.45	11.46	11.52	11.58	−0.09	0.12
RuO_4	12.96	12.94	12.91	12.97	13.05	13.15	0.10	0.60
$SOBr_2$	10.83	10.87	10.91	10.89	10.96	11.05	−0.03	0.06
$SPBr_3$	9.88	9.97	10.07	9.93	10.07	10.25	0.04	0.09
$SeCl_2$	9.50	9.60	9.70	9.56	9.69	9.87	0.14	0.01
SeO_2	12.21	12.22	12.24	12.24	12.31	12.39	0.11	0.20
$SiBrF_3$	12.24	12.27	12.30	12.31	12.36	12.43	−0.15	0.10
SiH_3I	10.23	10.26	10.30	10.29	10.29	10.31	−0.31	0.06
$SrBr_2$	10.10	10.12	10.15	10.16	10.22	10.29	−0.13	0.16
$SrCl_2$	10.57	10.69	10.84	10.58	10.78	11.06	0.04	0.14
SrI_2	9.31	9.35	9.43	9.38	9.33	9.27	−0.25	0.13
$TiBr_4$	10.90	10.94	10.97	10.99	11.05	11.12	−0.06	0.27
TiI_4	9.60	9.62	9.66	9.69	9.67	9.64	−0.22	0.23
$ZnBr_2$	11.11	11.14	11.17	11.19	11.25	11.32	−0.08	0.14
$ZnCl_2$	11.89	11.98	12.09	11.90	12.07	12.32	0.02	0.15
ZnF_2	14.50	14.46	14.41	14.52	14.56	14.60	0.12	0.38
ZnI_2	10.06	10.09	10.15	10.14	10.17	10.20	−0.33	0.11
$ZrBr_4$	11.10	11.13	11.18	11.18	11.24	11.32	−0.08	0.19
$ZrCl_4$	12.15	12.24	12.35	12.15	12.32	12.57	0.05	0.20
ZrI_4	9.81	9.83	9.87	9.88	9.88	9.88	−0.23	0.15

C Basis sets for BAND calculations

The basis set files used for the BAND calculations are TZ3P and QZ6P basis sets,[1] augmented with additional high-angular-momentum functions. The basis set files are provided as .txt files which list all exponents for each angular momentum.

References

(1) Förster, A.; Visscher, L. GW100: A Slater-Type Orbital Perspective. *J. Chem. Theory Comput.* **2021**, *17*, 5080–5097.
